# A combination of hard and soft templating for the fabrication of silica hollow microcoils with nanostructured walls

**DOI:** 10.1186/1556-276X-6-330

**Published:** 2011-04-13

**Authors:** Carlos Rodriguez-Abreu, Neus Vilanova, Conxita Solans, Masaki Ujihara, Toyoko Imae, Arturo López-Quintela, Seiji Motojima

**Affiliations:** 1 International Iberian Nanotechnology Laboratory (INL), Av. Mestre José Veiga, Braga, 4715-310, Portugal; 2 Instituto de Química Avanzada de Cataluña. Consejo Superior de Investigaciones Científicas (IQAC-CSIC), Jordi Girona 18-26, Barcelona, 08034, Spain; 3 Graduate Institute of Engineering, National Taiwan University of Science and Technology, 43 Keelung Road, Section 4, Taipei, Taiwan; 4 Departamento de Química Física, Facultad de Química, Universidad de Santiago de Compostela, Santiago de Compostela, 15782, Spain; 5 Toyota Physical & Chemical Research Institute, Nagakute, Aichi, 480-1192, Japan

## Abstract

Hollow silica microcoils have been prepared by using functionalized carbon microcoils as hard templates and surfactant or amphiphilic dye aggregates as soft templates. The obtained materials have been characterized by electron and optical microscopy, nitrogen sorption and small angle X-ray scattering. The obtained hollow microcoils resemble the original hard templates in shape and size. Moreover, they have mesoporous walls (pore size ≈ 3 nm) with some domains where pores are ordered in a hexagonal array, originated from surfactant micelles. The obtained silica microcoils also show preferential adsorption of cationic fluorescent dyes. A mechanism for the formation of silica microcoils is proposed.

## Introduction

The use of templates or scaffolds is one of the main strategies for the fabrication of advanced materials with new structures at the nano and micro scales that have attracted considerable research effort over the past decades. Templates can be classified as 'hard' and 'soft'. Hard templates are usually solid-state materials with particular structure and morphology, whereas soft templates are generally in a fluid-like state.

Hard templating is a conceptually straightforward and highly effective method to prepare hollow structures that mimic and/or complement the original shape of the templates [1,2], which usually consists of the following steps: 1. Preparation of hard templates; 2. Functionalization/modification of template surface; 3. Coating the templates with the target shell material; and 4. Selective removal of the templates to obtain hollow structures. Silica particles and polymer latex colloids belong to the group of materials commonly employed as hard templates.

On the other hand, soft templates such as supramolecular self-assemblies are a powerful tool for the bottom-up synthesis of nanomaterials [3-6], particularly mesoporous inorganic solids. In this approach, there is a cooperative interaction between self-assemblies and inorganic species that lead to structuration.

Carbon microcoils (CMCs) [7], with coil diameters in the order of micrometers, are a class of carbon materials with singular properties, such as mechanical elasticity [8], high hydrogen sorption [9] and electromagnetic wave absorption [10]. Carbon microcoils also help to improve material properties when incorporated in hybrid composites [11], or as template for other materials [12,13].The formation of a coiled structure is attributed to the fact that the crystal faces of the catalyst used for the synthesis show different activity (i.e. catalytic anisotropy) in terms of carbon growth [7].

Although there is some literature on the use of carbon nanotubes (CNT) as scaffolds for the preparation of silica nanotubes with different morphologies [14-16], carbon materials with a peculiar structure such as CMC has not been used in a combined hard and soft templating strategy to produce hierarchically ordered materials. In this context, we report the results on the use of such a method to fabricate nanoporous hollow silica microcoils and discuss the characterization of the obtained materials. The combination of soft and hard templating provides versatility for the preparation of materials with properties deriving from structuration at different scales.

## Experimental

### Materials

Carbon microcoils were synthesized according to a previous publication [7]. Hexadecyltrimethylammonium bromide (CTAB), tetraethylorthosilicate (TEOS), rhodamine B and fluorescein were supplied by Sigma-Aldrich (USA). A Perylenebis(dicarboximide) dye (referred herein as PDI) was synthesized according to the literature [17].Ultrapure water (resistivity = 18.2 MΩ/cm) was used in the experiments. All chemicals were used without further purification.

### Preparation of silica samples

Surface functionalization of CMCs was carried out by oxidation following a method already reported [18]. Functionalized CMCs with -COOH groups are referred herein as CMC-COOH. In a typical preparation of silica hollow coils by sol-gel reaction, CTAB or PDI is dissolved in NH_3 _(aq., 25%). Then, CMC-COOHs are dispersed in the mixture by ultrasonication. Finally, TEOS is added and the mixture is stirred with a magnetic stirring bar for 3 h at 70°C. The resulting precipitate is washed, filtered, dried and calcined in air for 6 h at 600°C (heating rate = 1°C/min), above the decomposition temperature of CMC-COOHs, as determined by thermogravimetric analysis.

### Characterization

Scanning electron microscopic (SEM) images were collected with a Hitachi TM-1000 (Japan) and with a Zeiss UltraPlus FESEM instrument (Germany). Transmission electron microscopic (TEM) images were taken with a Hitachi H-7000(Japan). Specimens were deposited on copper grids from ethanol dispersions. Fluorescence microscopic images were collected with a Nikon Eclipse TE2000-U(Japan); for the observation, samples were immersed in an aqueous dye solution for 1 h and then rinsed thoroughly to remove the non-adsorbed dye. Small angle X-ray scattering (SAXS) measurements were performed in an instrument equipped with a Kratky camera and a linear position sensitive detector, OED 50 M, both from MBraun (Austria). Measurements were carried out at 0.5 kW with radiation coming from a Siemens generator, model Krystalloflex 760 (Germany). Nitrogen sorption isotherms were determined using a Micromeritics TriStar 3000 instrument (USA). Samples were degassed at 200°C, and weighed prior to sorption experiments. The pore size distribution was determined by the Barret-Joyner-Halenda (BJH) method [19].

## Results and discussion

**Figure 1:**
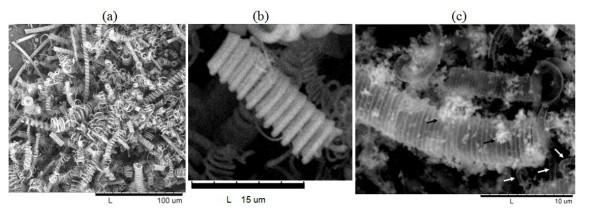
**SEM images (a, b) CMC-COOH before silica coating, (c) microcoil after silica-coating and calcination**. The initial weight ratios for the silica coating process were CTAB/NH_3_(aq.)/CMC-COOH/TEOS = 13.9/70.4/0.6/15.1. The black arrows indicate silica particles adhered to the coils whereas white arrows indicate translucid coil sections.

**Figure 2:**
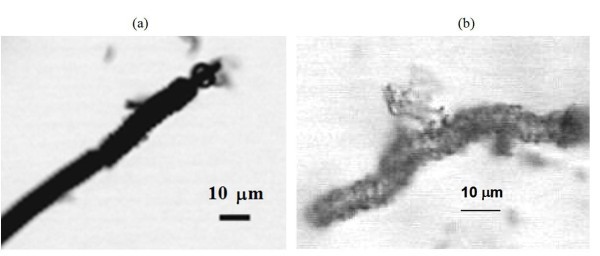
**Optical microscopic images of (a) CMC-COOH and (b) hollow silica microcoil prepared using CTAB**.

As can be seen in Figure 1a,b, the CMC-COOHs used as templates are polydisperse in diameter, pitch and length; some of them are hundreds of micrometers in length. During the sol-gel reaction, the surface of the CMC-COOHs is covered by a silica deposit. After calcination, i.e. after removal of CMC-COOHs, silica coils are left (see Figure 1c). Some sections of the coils seem more transparent, due to their very thin silica walls, while coil size and morphology appear similar to that of original CMC-COOHs. Silica particles can also be observed on the surface of the coils. The differences in contrast of the two specimens (CMC-COOHs and silica coils) can also be clearly observed by the optical microscope (Figure 2). The images are a proof that the CMC-COOHs used as templates have been burnt off and silica coils are left over, although also non-CMC-templated silica particles are obtained mixed with the coils, as isolated silica particles can also form in the bulk solution during the sol-gel reaction. The amount of non-CMC-templated silica decreases by reducing the CTAB/CMC-COOH ratios, so that most of CTAB is adsorbed on the CMC-COOH surface.

**Figure 3:**
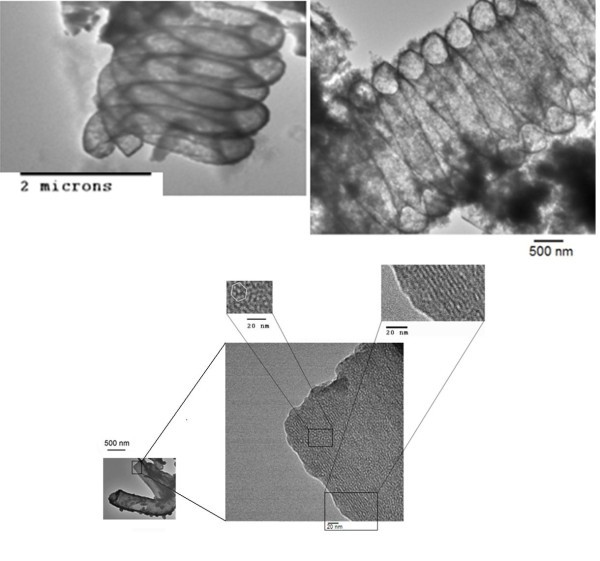
**TEM images of different sections of silica hollow microcoils**. The initial weight ratios for the silica coating process were CTAB/NH_3_(aq.)/CMC-COOH/TEOS = 13.7/69.4/2.0/14.9.

TEM images of silica hollow microcoils are presented in Figure 3. The photographs at low magnification show unambiguously the hollow nature of the specimens; the very thin silica walls allow the transmission of the electron beam. The fine structure of the walls can be imaged at higher magnification. Arranged channels of about 3-nm width were observed in some sections of the microcoil walls. The cross section of those channels is circular, namely, the walls contain cylindrical mesopores, some of which ordered in a hexagonal fashion. However, it should be pointed out that mesopores with disordered domains (worm-hole morphology) also exist in the microcoil walls. The existence of mesopores was confirmed by nitrogen sorption experiments, which gave a relatively narrow pore size distribution with a maximum at 2.3 nm, in agreement with TEM observations (see Figure S1 in Additional file 1). Samples of silica coils were also analyzed by SAXS. Patterns showed a strong peak corresponding to a Bragg spacing *d *of 3.8 nm (see Figure S2 in Additional file 1); the lattice parameter for a hexagonal array (*a *= 2*d*/√3) was calculated as 4.4 nm, which is similar to that of MCM-41 silica [20].

**Figure 4:**
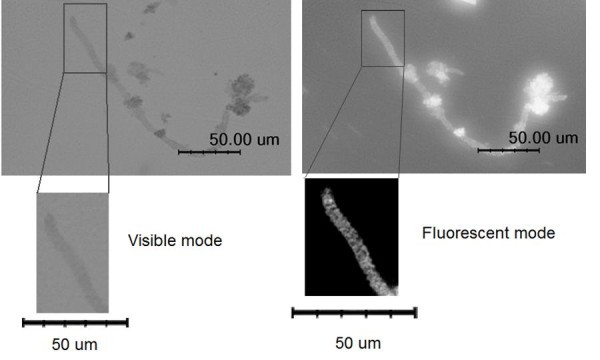
**Visible and fluorescent microscopic images of hollow silica microcoils prepared using CTAB after soaking them in an aqueous solution of rhodamine B**.

The dye adsorption properties of silica hollow microcoils were tested. As can be seen in Figure 4, the microcoils pre-soaked in a cationic dye (rhodamine B) solution are fluorescent namely, the dye strongly adsorbs on the surface of silica microcoil, indicating that the surface is negatively charged, as expected from the high-pH synthesis conditions. On the other hand, no fluorescence was emitted from a sample pre-soaked with an anionic dye (e.g. fluorescein), since there is no charge matching and hence no adsorption of the anionic dye on the microcoils.

**Figure 5:**
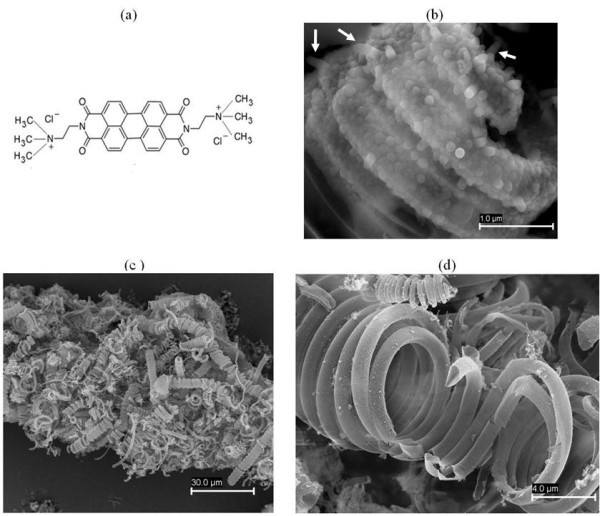
**Silica microcoils prepared using PDI**. **(a) **Molecular structure of PDI. **(b) **SEM image of silica microcoils at PDI/CMC-COOH ratio = 3; the arrows indicate elongated particles adhered to the coils. **(c, d) **SEM image of silica microcoils at PDI/CMC-COOH ratio = 0.2. The other initial weight ratios for the silica coating process were NH_3_(aq.)/CMC-COOH/TEOS = 300/0.37/10.1.

We also found that PDI (see Figure 5a), which is known to self-assemble in water [17], can be used as a soft template for the silica coating of CMC-COOHs. PDI has n-type semiconductor properties, with possible applications in organic field effect transistors and photovoltaics. Moreover, PDI molecules adsorb strongly to the CMC-COOH surface, as confirmed by UV-vis spectroscopy (see Figure S3 in Additional file 1). In the absence of CMC-COOH, PDI induces the formation of elongated silica particles in the sol-gel reaction mixture, which is attributed to the templating effect of cylindrical self-assemblies of the dye in solution [17]. At high PDI/CMC-COOH ratios, those elongated silica particles, precipitated from the solution, coat the surface of CMC-COOHs (see Figure 5b). When the TEOS/CMC-COOH ratio is decreased, hollow silica microcoils with smoother surface are obtained, suggesting that silica formation on the CMC-COOH surface is favoured over that occurring in the bulk solution (see Figure 5c,d). The yield of silica microcoils relative to amorphous silica also increases when the PDI/CMC-COOH ratios are decreased. It is to be noted here that when PDI is used as template, the walls of silica coils are probably microporous rather than mesoporous as suggested by preliminary nitrogen sorption measurements, which gave isotherms with features typical of microporous solids.

The fluorescence emission properties of the PDI adsorbed on CMC-COOH were preserved after silica coating. Dispersions of the PDI-silica coils in ethanol gave two emission bands at 540 and 575 nm (see Figure S4 in Additional file 1). However, a change in the UV-vis spectrum was observed (see Figure S5 in Additional file 1). Neat PDI solutions in ethanol show two absorption maxima at 530 and 590 nm, whereas the dispersions of PDI-silica coils exhibited only one maximum at 520 nm and a shoulder at about 580 nm.

**Figure 6:**
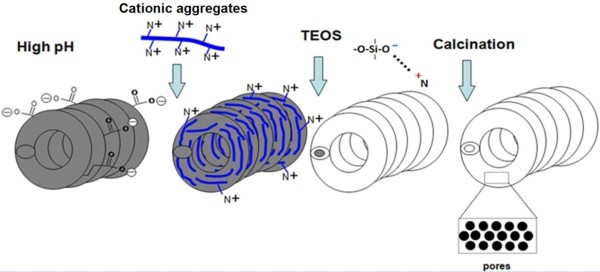
**Proposed scheme for the mechanism of formation of hollow silica microcoils**.

Based upon the experimental evidence, a mechanism for the formation of silica hollow microcoils with mesoporous walls can be proposed (see Figure 6). Cationic aggregates, which are expected to be elongated at the used concentrations of amphiphile and electrolyte [17,21,22], adsorb on the surface of negatively-charged CMC-COOHs. When TEOS is added at high pH, the silica coating is built up through anchoring of silica via electrostatic interaction of siloxy ions with ammonium ions of the amphiphilic molecules adsorbed on CMCs. During the sol-gel reaction, free cationic aggregates are also cooperatively incorporated as porogens in the polysiloxane gel. At this stage, there might be some preferential orientation of aggregates in the silica layers. Depending on amphiphile concentration, excess silica particles with inner mesostructure forms in the bulk solution and some of those particles also adhere to the silica layers on the surface of CMCs. Finally, upon calcination, the CMCs (hard templates) and amphiphilic molecules (soft templates) are burnt off, and silica hollow microcoils with porous walls are obtained.

## Summary

Hollow silica microcoils with mesostructured walls were prepared by using carbon microcoils and amphiphilic molecules as hard and soft templates, respectively, and both serve as porogens upon calcination. Cationic aggregates adsorb on functionalized CMCs and behave both as an anchor and porogen of silica. The mesopores originated from surfactant aggregates were either ordered hexagonally or had a disordered, worm-hole morphology. Since the obtained hollow silica microcoils have a negatively charged surface (as a result of synthesis conditions), they show advantages for preferentially trapping cationic molecules. The method described here can be used to prepare hollow microcoils of other oxides via sol-gel reaction.

## Abbreviations

CMCs: carbon microcoils; CNT: carbon nanotubes; CTAB: hexadecyltrimethylammonium bromide; PDI: Perylenebis(dicarboximide); SAXS: small angle X-ray scattering; SEM: scanning electron microscopic; TEM: Transmission electron microscopic; TEOS: tetraethylorthosilicate.

## Competing interests

The authors declare that they have no competing interests.

## Authors' contributions

CR-A conceived the study and participated in its design and coordination, as well as in sample preparation and characterization by TEM, SEM and SAXS. NV participated in TEM observations and in spectroscopic measurements. CS, AL-Q and TI participated in the preparation and revision of the manuscript as well as in giving access to SAXS, SEM and TEM facilities. MU participated in electronic and optical microscopy experiments. SM carried out the synthesis of carbon microcoils. All authors read and approved the final manuscript.

## Supplementary Material

Additional file 1 **Figure S1**. Pore size distribution of silica hollow microcoils. The initial weight ratios for preparation were CTAB/NH_3_(aq.)/CMC-COOH/TEOS = 13.9/70.4/0.6/15.1. The distribution is estimated from nitrogen sorption measurements using the Barret-Joyner-Halenda (BJH) method. **Figure S2**. SAXS spectra of (a) CMC-COOH, (b) hollow silica microcoils prepared with initial CTAB/NH_3_(aq.)/CMC-COOH/TEOS weight ratios of 13.9/70.4/0.6/15.1. The numbers indicate the peak position ratios corresponding to a hexagonal lattice. (c) Hollow silica microcoils prepared with initial CTAB/NH_3_(aq.)/CMC-COOH/TEOS weight ratios of 8.1/85.0/2.4/4.5. **Figure S3**. UV-vis spectra of PDI aqueous solutions before contact (continuous line) and after 1 h contact (dashed line) with CMC-COOHs. The decrease in absorbance is due to adsorption of PDI molecules on the surface of CMC-COOHs. **Figure S4**. Fluorescence emission spectrum of of PDI on CMC-COOH after silica coating. The spectrum corresponds to a dispersion in ethanol measured in a 1-cm path length cuvette. **Figure S5**. UV-vis absorption spectra of neat PDI (continuous line) and PDI on CMC-COOH after silica coating (dashed line). Spectra correspond to dispersions in ethanol measured in 1-cm path length cuvettes. Click here for file
