# Supplementary material for: A combination of hard and soft templating for the fabrication of silica hollow microcoils with nanostructured walls
Source: Nanoscale Res Lett. 2011 Apr 13;6(1):330. doi: 10.1186/1556-276X-6-330 (PMC3211418; doi:10.1186/1556-276X-6-330)
Supplement: Additional file 1 — Figure S1. Pore size distribution of silica hollow microcoils. The initial weight ratios for preparation were CTAB/NH3(aq.)/CMC-COOH/TEOS = 13.9/70.4/0.6/15.1. The distribution is estimated from nitrogen sorption measurements using the Barret-Joyner-Halenda (BJH) method. Figure S2. SAXS spectra of (a) CMC-COOH, (b) hollow silica microcoils prepared with initial CTAB/NH3(aq.)/CMC-COOH/TEOS weight ratios of 13.9/70.4/0.6/15.1. The numbers indicate the peak position ratios corresponding to a hexagonal lattice. (c) Hollow silica microcoils prepared with initial CTAB/NH3(aq.)/CMC-COOH/TEOS weight ratios of 8.1/85.0/2.4/4.5. Figure S3. UV-vis spectra of PDI aqueous solutions before contact (continuous line) and after 1 h contact (dashed line) with CMC-COOHs. The decrease in absorbance is due to adsorption of PDI molecules on the surface of CMC-COOHs. Figure S4. Fluorescence emission spectrum of of PDI on CMC-COOH after silica coating. The spectrum corresponds to a dispersion in ethanol measured in a 1-cm path length cuvette. Figure S5. UV-vis absorption spectra of neat PDI (continuous line) and PDI on CMC-COOH after silica coating (dashed line). Spectra correspond to dispersions in ethanol measured in 1-cm path length cuvettes. [file 1556-276X-6-330-S1.DOC]

**Supplementary Information**

**
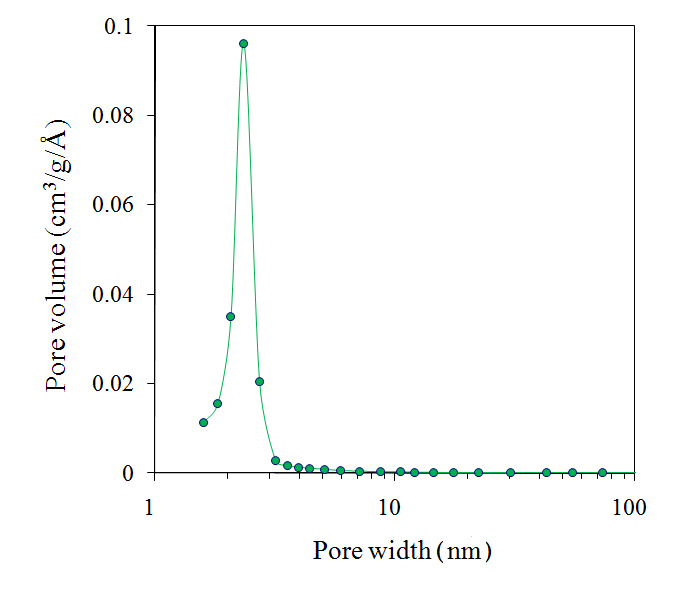
**

**Figure S1:** Pore size distribution of silica hollow microcoils. The initial weight ratios for preparation were CTAB/NH_3_(aq)/CMC-COOH/TEOS=13.9/70.4/0.6/15.1. The distribution is estimated from nitrogen sorption measurements using the Barret-Joyner-Halenda (BJH) method.


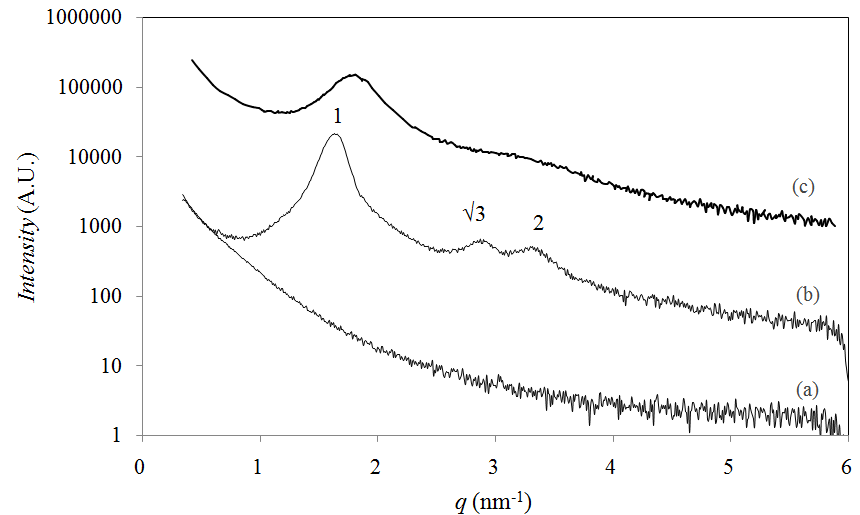


**Figure S2:** SAXS spectra of (a) CMC-COOH (b) Hollow silica microcoils prepared with initial CTAB/NH3(aq)/CMC-COOH/TEOS weight ratios of 13.9/70.4/0.6/15.1 . The numbers indicate the peak position ratios corresponding to a hexagonal lattice. (c) Hollow silica microcoils prepared with initial CTAB/NH3(aq)/CMC-COOH/TEOS weight ratios of 8.1/85.0/2.4/4.5

**
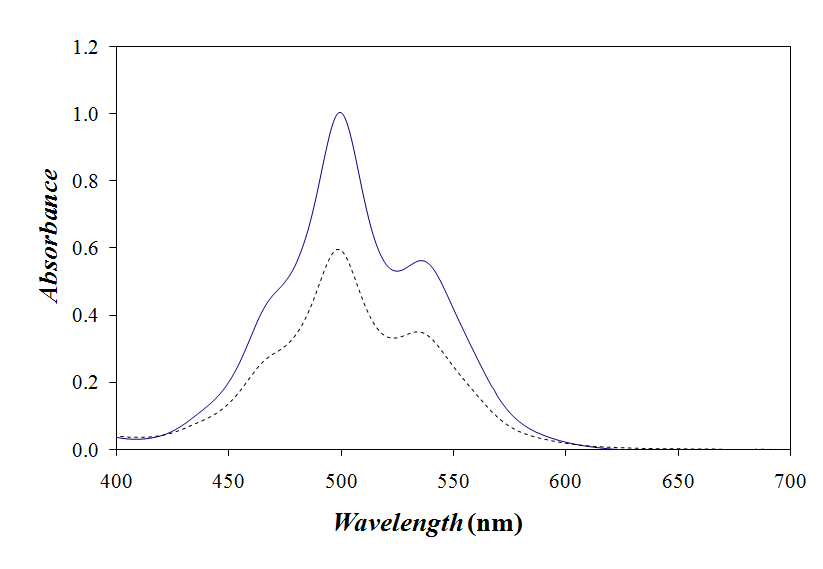
**

**Figure S3:** UV-vis spectra of PDI aqueous solutions before contact (continuous line) and after 1 h contact (dashed line) with CMC-COOHs. The decrease in absorbance is due to adsorption of PDI molecules on the surface of CMC-COOHs.

**Figure S4:** Fluorescence emission spectrum of of PDI on CMC-COOH after silica coating. The spectrum corresponds to a dispersion in ethanol measured in a1-cm path length cuvette.

**Figure S5:** UV-Vis absorption spectra of neat PDI (continuous line) and PDI on CMC-COOH after silica coating (dashed line). Spectra correspond to dispersions in ethanol measured in 1-cm path length cuvettes.
